# Circulating musclin as a potential prognostic biomarker for major adverse cardiovascular events in surgically treated acute aortic dissection: a retrospective cohort study

**DOI:** 10.3389/fcvm.2026.1953235

**Published:** 2026-10-05

**Authors:** Jia Liu, Zhihui Liu, Liang Guo, Zhiyuan Wang, Jinfang Wu, Xiaobing Li, Huijun Zhang, Huining Liu

**Affiliations:** 1 Department of Cardiac Surgery, The First Hospital of Hebei Medical University, Shijiazhuang, Hebei, China; 2 School of Nursing, Hebei Medical University, Shijiazhuang, Hebei, China; 3 Endoscopy Center, The First Hospital of Hebei Medical University, Shijiazhuang, Hebei, China; 4 Department of Thoracic Surgery, The First Hospital of Hebei Medical University, Shijiazhuang, Hebei, China

**Keywords:** acute aortic dissection, cardiac surgical procedures, major adverse cardiovascular events, musclin, prognosis, risk stratification

## Abstract

**Background:**

Musclin is a skeletal muscle-derived myokine with reported cardioprotective effects; however, its prognostic relevance in acute aortic dissection (AAD) remains uncertain. This study examined whether admission Musclin levels were associated with postoperative major adverse cardiovascular events (MACE) and whether they added prognostic information beyond conventional clinical indicators.

**Methods:**

We retrospectively enrolled 108 surgically treated AAD patients from 2022 to 2023. Musclin levels were measured by ELISA in residual serum samples collected at admission. The primary endpoint was composite MACE. Kaplan–Meier survival analysis, multivariate Cox regression, time-dependent ROC, IDI and NRI were used to evaluate the predictive and incremental value of Musclin.

**Results:**

During 400-day follow-up, 36 patients (33.3%) developed MACE. The CART-derived optimal Musclin cutoff was 4.815 ng/mL. Kaplan–Meier analysis showed that low Musclin group had significantly higher MACE cumulative incidence than high Musclin group (48.2% vs. 17.3%, log-rank *P* < 0.001). Multivariate Cox regression confirmed circulating Musclin associated with a lower risk of MACE (HR = 0.314, 95%CI: 0.104–0.943, *P* = 0.039). Time-dependent AUC analysis demonstrated that the combined model incorporating Musclin (model 2) achieved superior AUC compared with the clinical model (model 1). Even at the 250-day time point, where the incremental gain (*Δ*AUC) was minimal, the combined model still exhibited significantly better discriminative performance (0.876 vs. 0.837, DeLong *P* = 0.013). At the 250-day landmark, IDI was 0.153 (95%CI: 0.018–0.286, *P* = 0.012) and overall NRI was 0.167 (95%CI: 0.003–0.336, *P* = 0.044), with event-specific NRI reaching 0.361 (*P* = 0.012). Sensitivity analyses yielded directionally consistent associations when Musclin was analyzed using the cohort median or as a continuous variable.

**Conclusions:**

In this single-center retrospective cohort, lower admission Musclin was associated with a higher risk of postoperative MACE. Musclin may be a severity-related biomarker that could complement conventional risk indicators, but its independent clinical utility, optimal threshold, and role in risk stratification require prospective multicenter validation with comprehensive perioperative adjustment before clinical use.

## Introduction

Acute aortic dissection (AAD) is an extremely lethal emergency of large-vessel disease characterized by sudden tearing of the aortic intima, formation of a false lumen, and systemic multi-organ hypoperfusion, carrying devastating short- and medium-term postoperative adverse event risks (1). Despite remarkable advances in surgical techniques, perioperative intensive care and standardized antihypertensive regimens, patients with AAD still face a high risk of Major Adverse Cardiovascular Events (MACE) during follow-up, including all-cause death, aorta-related reoperation, stroke, and readmission, which seriously threaten patients’ long-term quality of survival (2). Conventional preoperative risk indicators, including hypertension, NT-proBNP, coagulation profiles and left ventricular ejection fraction, partially reflect cardiac strain and systemic hypercoagulability, yet they fail to capture skeletal muscle-cardiovascular crosstalk, an emerging pathophysiological axis that mediates perioperative multi-organ injury (3). Accurate preoperative risk stratification is urgently required to identify high-risk patients for intensified monitoring, personalized blood pressure control and aggressive organ protection; however, current routine laboratory biomarkers lack specific information linking skeletal muscle function to aortic adverse outcomes, highlighting an unmet clinical need for novel predictive myokines.

Musclin, alternatively termed osteocrin (Ostn), is a skeletal muscle-secreted peptide classified as a myokine belonging to the muscle-specific cytokine family (4). Serving as an endogenous ligand for the natriuretic peptide clearance receptor NPR3, Musclin competitively binds to this receptor to elevate bioactive levels of atrial natriuretic peptide (ANP) and B-type natriuretic peptide (BNP) in peripheral circulation (5). Originally, Musclin was predominantly investigated for its regulatory effects on metabolic diseases including obesity and type 2 diabetes (4). Accumulating preclinical evidence has further uncovered its vital biological functions within the cardiovascular system. Mechanistically, Musclin modulates vascular homeostasis and structural remodeling via regulating calcium channel activity and suppressing excessive proliferation of vascular smooth muscle cells (VSMCs) (6). The pathological progression of acute aortic dissection (AAD) is fundamentally attributed to VSMC apoptosis, excessive activation of matrix metalloproteinases, and progressive degradation of aortic extracellular matrix (7). Musclin is hypothesized to participate in the modulation of aortic wall remodeling throughout the initiation and advancement of AAD. Multiple clinical observational studies have validated that decreased circulating Musclin concentrations are closely correlated with cardiac dysfunction, atrial fibrillation and chronic heart failure, which supports its utility as a circulating biomarker reflecting cardiac loading status and systemic multi-organ injury burden (8). In addition, sarcopenia and impaired skeletal muscle function have been proven to independently predict early postoperative mortality following AAD surgical repair, highlighting the prognostic significance of skeletal muscle-derived myokines for perioperative risk stratification (9). However, the prognostic association between circulating Musclin and postoperative MACE in patients with AAD has not been established. Furthermore, whether Musclin could yield incremental prognostic information independent of conventional clinical risk factors has not yet been elucidated.

In this retrospective cohort of surgically treated AAD patients, we evaluated the association between admission serum Musclin and postoperative MACE during 400 days of follow-up. We also explored whether Musclin added prognostic information beyond conventional clinical indicators. The CART-derived threshold was treated as exploratory because it was derived and assessed within the same cohort.

## Materials and methods

### Patients

This single-center retrospective observational cohort consecutively enrolled 108 patients diagnosed with surgically treated acute aortic dissection (AAD) admitted to our institution between January 2022 and December 2023. Comprehensive clinical information was systematically extracted from electronic medical records, encompassing demographic characteristics, anthropometric indices, medical history of cardiovascular comorbidities, perioperative laboratory parameters, and transthoracic echocardiography measurements. Laboratory indicators included liver and renal function, serum lipid profiles, routine coagulation parameters, cardiac troponin, high-sensitivity C-reactive protein (hs-CRP), and N-terminal pro-B-type natriuretic peptide (NT-proBNP). This study complied with the Declaration of Helsinki and was approved by the Ethics Committee Review Board of Hebei Medical University; informed consent was waived due to the retrospective nature of data collection.

### Patients follow-up

This study conducted regular follow-up of discharged patients through multiple methods, including the hospital electronic medical record system, outpatient revisit records, telephone interviews, and WeChat communication. The follow-up starting point was the day of surgical discharge, and the endpoint was the first occurrence of MACE, all-cause death, or the follow-up deadline (400 days post-surgery), whichever occurred first. The primary composite endpoint (MACE) was defined as all-cause death, aorta-related reoperation, stroke, and aorta-related readmission. During the follow-up period, contact was made with patients or their immediate family members every 3 months via telephone or WeChat to inquire about survival status, symptom changes, and rehospitalization; meanwhile, outpatient revisit data and local hospital visit records were combined to verify and confirm reported events. All endpoint events were adjudicated by two independent cardiovascular specialists based on original medical records, and consensus was reached through discussion and consultation when opinions differed.

### Measurement of circulating musclin

This study utilized residual serum samples collected upon admission before surgery, which were immediately aliquoted into multiple small-volume tubes and stored stably at −80 °C to avoid repeated freeze-thaw cycles. All samples were assayed in a single uniform batch after the completion of the 400-day follow-up using a commercially available human Musclin enzyme-linked immunosorbent assay (ELISA) kit (Cusabio, CSB-EL017269MO). All experimental procedures were strictly implemented following the manufacturer's standard protocols. Laboratory technicians performing the ELISA measurements were fully blinded to patients’ baseline clinical characteristics and follow-up MACE outcomes. Each serum sample was measured in technical duplicate wells, and the mean absorbance value of two replicates was adopted as the final circulating Musclin concentration to reduce intra-assay measurement error. Although retrospective batch testing of archived frozen serum may introduce minor pre-analytical deviations related to long-term cryopreservation, all samples underwent identical storage, aliquoting, and testing procedures, which ensures consistent experimental conditions across the entire cohort and minimizes bias for intergroup comparisons.

### Statistical analysis

Data analysis was performed using SPSS 26.0 and R software (version 4.1.2). Continuous variables were first assessed for normality using the Shapiro–Wilk test. Normally distributed variables were expressed as mean ± standard deviation, and comparisons between two groups were performed using the t-test; non-normally distributed variables were expressed as median (P25, P75), and comparisons between two groups were performed using the Mann–Whitney U test. Categorical variables were expressed as number (%) and compared between groups using the *χ*^2^ test.

The CART algorithm was used to determine the optimal cutoff value of Musclin for predicting MACE. Because only 36 MACE events occurred, no data-splitting validation was performed. The CART threshold and all model-performance estimates were therefore treated as exploratory. Sensitivity analyses evaluated Musclin using the cohort median and as a continuous variable; these analyses assessed consistency of association but were not considered validation of the CART-derived threshold. Survival curves were plotted using the Kaplan–Meier method, and differences between groups were compared using the log-rank test. Univariate Cox regression was used to screen candidate variables (*P* < 0.20 was included in the multivariate analysis), and two models were constructed: Model 1 (clinical model) and Model 2 (clinical model + Musclin), with results expressed as HR and 95% CI.

Time-dependent ROC was used to evaluate model discrimination, and the DeLong test was used to compare differences in AUC; -2LL was used to evaluate model goodness-of-fit, and IDI and NRI were calculated at the time when *Δ*AUC was minimal to assess incremental predictive value. The predictive value of Musclin for MACE was evaluated through sensitivity analysis. All tests were two-sided, and *P* < 0.05 was considered statistically significant.

## Results

### Patient outcomes and clinical characteristics

The primary composite endpoint of this study was defined as major adverse cardiovascular events (MACE) occurring within 400 days after surgery, encompassing all-cause death, aorta-related reoperation, stroke, and aorta-associated readmission. The total cohort consisted of 108 surgically treated AAD patients, among whom 36 (33.3%) developed composite MACE and 72 (66.7%) remained event-free. Among 36 MACE events, 12 cases were all-cause death (11.1%), 8 were aorta-related reoperations (7.4%), 6 were strokes (5.6%), and 10 were aorta-associated readmissions (9.3%). Baseline demographic, clinical and laboratory variables were compared between MACE and non-MACE groups (Table 1).

**Table 1:** Baseline clinical characteristics stratified by postoperative MACE status.

Variable	MACE (*n* = 36)	Non-MACE (*n* = 72)	t/*χ*2/Z	*P* value
Age	61.0 (56.5, 70.5)	54.5 (44.3, 61.8)	−3.355	＜0.001
Gender			0.329	0.566
Male	25 (35.2%)	46 (64.8%)		
Female	11 (29.7%)	26 (70.3%)		
HBP			3.342	0.068
YES	32 (88.9%)	53 (73.6%)		
NO	4 (11.1%)	19 (26.4%)		
BMI	27.34 ± 3.32	26.41 ± 3.82	1.238	0.218
ALT	21.90 (17.0, 58.15)	18.00 (13.40, 31.85)	−2.219	0.026
AST	26.35 (19.83, 64.60)	21.40 (19.03, 25.40)	−2.473	0.013
Alb	39.60 ± 4.41	39.48 ± 4.62	0.130	0.897
BUN	5.12 (3.696, 7.03)	6.52 (5.05, 7.57)	−0.792	0.428
CR	87.85 (66.85, 111.725)	78.65 (65.20, 97.075)	−1.154	0.249
FN	2.57 (1.8325, 4.723)	2.51 (2.0025, 3.37)	−0.525	0.600
DD	7.68 (2.5525, 36.52)	8.66 (4.01, 35.57)	−0.192	0.848
NT-proBNP	348.50 (179.75, 669.75)	128.00 (80.25, 207.50)	−4.305	＜0.001
musclin	2.67 (1.97, 4.65)	5.46 (3.89, 6.80)	−4.917	＜0.001

HBP, hypertension; ALT, alanine aminotransferase; AST, aspartate aminotransferase; Alb, albumin; BUN, blood urea nitrogen; CR, creatinine; DD, D-Dimer; FN, fibrinogen; NT-proBNP, N-terminal pro-B-type natriuretic peptide.

Patients who experienced MACE were significantly older (median age: 61.0 vs. 54.5 years, *P* < 0.001). No intergroup differences were observed in sex distribution or hypertension prevalence (all *P* > 0.05). Body mass index and serum albumin levels were comparable across the two groups (*P* > 0.05).

For liver injury biomarkers, MACE patients exhibited markedly elevated alanine aminotransferase (ALT, *P* = 0.026) and aspartate aminotransferase (AST, *P* = 0.013), indicating more severe preoperative hepatic hypoperfusion injury. Renal markers (blood urea nitrogen, creatinine), fibrinogen and D-dimer showed no significant between-group disparities (all *P* > 0.05). Notably, the MACE subgroup displayed substantially higher NT-proBNP (median: 348.50 vs. 128.00, *P* < 0.001), reflecting heavier preoperative cardiac overload.

Circulating Musclin concentrations differed between groups: the median Musclin level in MACE patients was only 2.67 ng/mL, far lower than the 5.46 ng/mL recorded in event-free patients (*P* < 0.001). This baseline association suggested that reduced admission Musclin may mark a higher postoperative MACE risk. Given the significant difference in circulating Musclin levels between MACE and non-MACE patients at baseline, we next sought to establish an optimal cutoff value for clinical risk stratification.

### Risk stratification for MACE based on the CART-derived musclin cutoff

To translate circulating Musclin into a clinically applicable risk-stratification tool for prognostic stratification of postoperative MACE, we employed a classification and regression tree (CART) algorithm to determine the optimal threshold for predicting postoperative MACE. Decision tree analysis identified the optimal cutoff value of circulating Musclin as 4.815 ng/mL. Patients were accordingly categorized into the low Musclin group (Musclin < 4.815 ng/mL, *n* = 56) and the high Musclin group (Musclin ≥ 4.815 ng/mL, *n* = 52) (Figure 1A). Kaplan–Meier analysis demonstrated that the cumulative incidence of MACE was markedly higher in the low Musclin group relative to the high Musclin group (48.2% versus 17.3%, log-rank *P* < 0.001) (Figure 1B), indicating that patients with admission Musclin below this threshold faced a nearly three-fold higher risk of subsequent adverse events.

**Figure 1:**
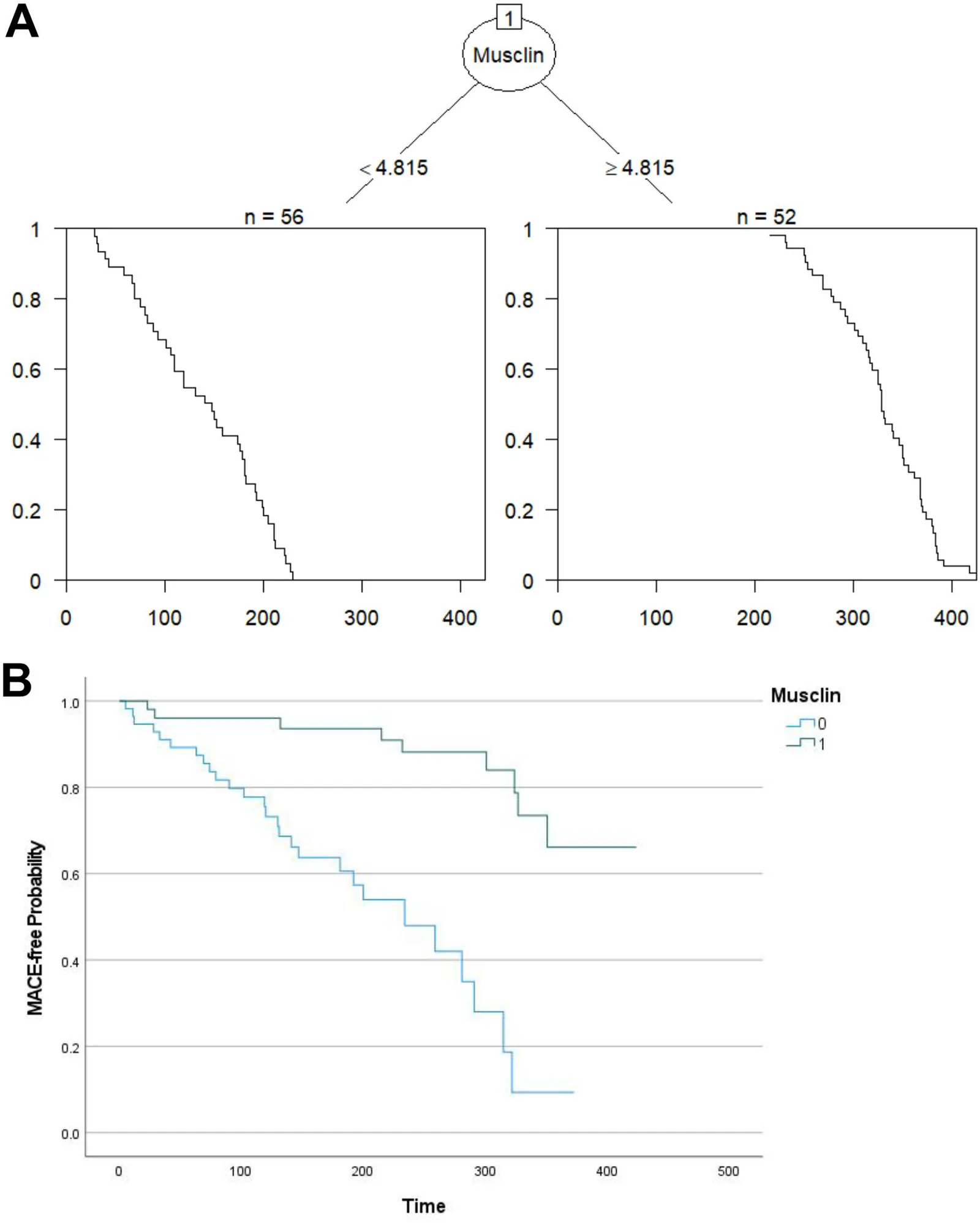
Kaplan–meier curves stratified by CART-derived musclin cutoff value. **(A)** CART analysis yielded an optimal Musclin cutoff of 4.815 ng/mL, separating patients into low Musclin (*n* = 56) and high Musclin (*n* = 52) groups, with individual survival curves displayed respectively. **(B)** Combined survival curves of two subgroups. Significant differences in MACE-free survival were confirmed by Log-rank test (*P* < 0.001).

### Clinical correlates of circulating musclin

To further explore the biological context underlying the prognostic value of Musclin, we examined its correlations with conventional laboratory parameters using Pearson correlation analysis. As illustrated in the correlation heatmap, serum Musclin concentration exhibited a moderate negative correlation with NT-proBNP (r = −0.456), blood urea nitrogen (BUN, r = −0.326), aspartate aminotransferase (AST, r = −0.327) and alanine aminotransferase (ALT, r = −0.214). Conversely, Musclin displayed a moderate positive correlation with left ventricular ejection fraction (EF, r = 0.414) (Figure 2).

**Figure 2:**
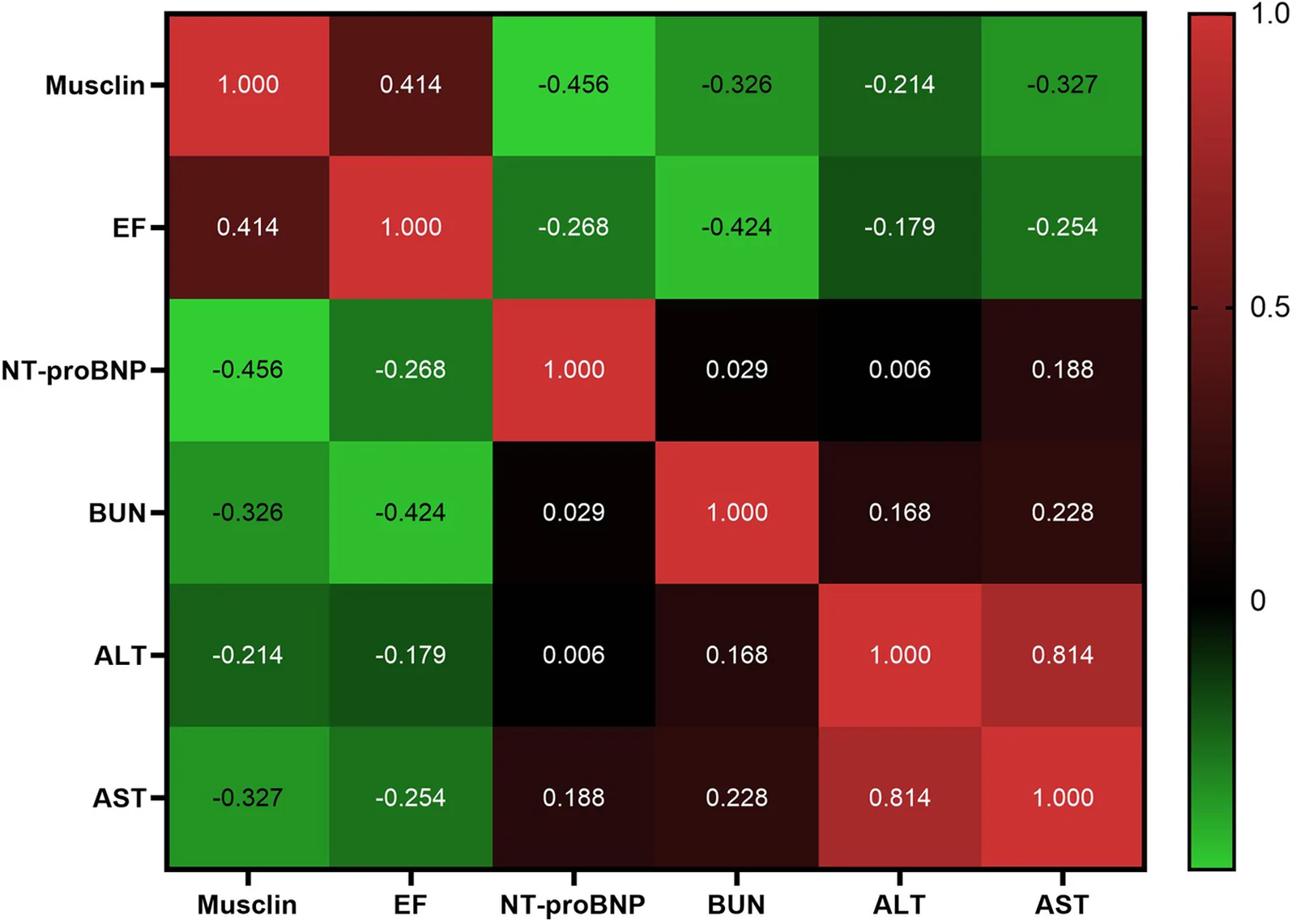
Clinical correlates of circulating musclin. The color of each block corresponds to the Pearson correlation coefficient. Red indicates a positive correlation between two variables, while green indicates a negative correlation. The number within each block represents the exact correlation coefficient. The color bar on the right ranges from 0 to 1; larger absolute values indicate stronger linear associations, whereas values near 0 suggest weak linear association. EF, left ventricular ejection fraction; NT-proBNP, N-terminal pro-B-type natriuretic peptide; BUN, blood urea nitrogen; ALT, alanine transaminase; AST, aspartate transaminase.

This correlation profile suggests that lower circulating Musclin is associated with worse cardiac functional status, higher NT-proBNP, and abnormalities in hepatic and renal markers. Accordingly, Musclin may partly reflect the severity of cardiac dysfunction and systemic illness.

### Association between circulating musclin and postoperative MACE

After 400 days of follow-up, we defined MACE as the outcome variable and follow-up time as the time variable. In univariable Cox regression, higher Musclin status was associated with a lower hazard of MACE (HR = 0.104, 95% CI: 0.043–0.255, *P* < 0.001). We next examined whether this association persisted after adjustment for the prespecified clinical and laboratory variables available in this retrospective dataset.

To further explore the independent predictive value of high Musclin for MACE risk, multivariate Cox regression was performed. Two multivariate Cox proportional hazards regression models were established using backward conditional selection to determine whether Musclin independently predicted MACE. Model 1 (clinical model) incorporated conventional risk factors: age (HR = 1.052), ALT (HR = 1.009), PT (HR = 1.361), and NT-proBNP (HR = 1.004) (Table 2).

**Table 2:** Multivariate cox proportional hazards regression analysis for model 1 predicting MACE.

Variable	β	SE	Wald χ^2^	df	*P* value	HR	95% CI for HR
Age	0.051	0.020	6.380	1	0.012	1.052	1.011–1.094
ALT	0.009	0.003	10.160	1	0.001	1.009	1.003–1.015
PT	0.308	0.157	3.841	1	0.050	1.361	1.000–1.853
NT-proBNP	0.004	0.001	36.162	1	<0.001	1.004	1.003–1.005

ALT, alanine aminotransferase; PT, Prothrombin Time; NT-proBNP, N-terminal pro-B-type natriuretic peptide.

Model 2 (clinical model + Musclin) further added circulating high Musclin into all covariates of Model 1. After full multivariate adjustment, high Musclin status remained associated with a reduced MACE risk (HR = 0.314, 95%CI: 0.104–0.943, *P* = 0.039). Given its correlations with NT-proBNP and EF, this association may partly reflect Musclin's role as an integrative marker of disease severity. In the multivariate model containing dichotomized Musclin, age, ALT, PT and NT-proBNP were all confirmed as independent adverse predictors for 400-day postoperative MACE, with corresponding HRs (95% CIs) of 1.043 (1.000–1.087), 1.007 (1.001–1.013), 1.363 (1.011–1.837) and 1.003 (1.002–1.005), respectively (Table 3, Figure 3). A visual prognostic nomogram integrating the five independent predictors was established to individually calculate the 100-day, 200-day, 300-day and 400-day MACE-free survival probability for each patient (Figure 4). The model exhibited excellent discriminative capacity, with a Harrell's C-index of 0.8588 (SE = 0.0285), indicating reliable risk stratification performance. These findings suggest that Musclin may provide additional prognostic information beyond the clinical variables included in the present model.

**Table 3:** Multivariate cox proportional hazards regression analysis for model 2 predicting MACE.

Variables	β coefficient	Standard Error	Wald χ^2^	df	*P*-value	HR [Exp(B)]	95% CI for HR
Age	0.042	0.021	3.832	1	0.05	1.043	1.000–1.087
ALT	0.007	0.003	5.524	1	0.019	1.007	1.001–1.013
PT	0.310	0.152	4.133	1	0.042	1.363	1.011–1.837
NT-proBNP	0.003	0.001	23.38	1	<0.001	1.003	1.002–1.005
Musclin group	−1.159	0.561	4.265	1	0.039	0.314	0.104–0.943

ALT, alanine aminotransferase; PT, Prothrombin time; NT-proBNP, N-terminal pro-B-type natriuretic peptide.

**Figure 3:**
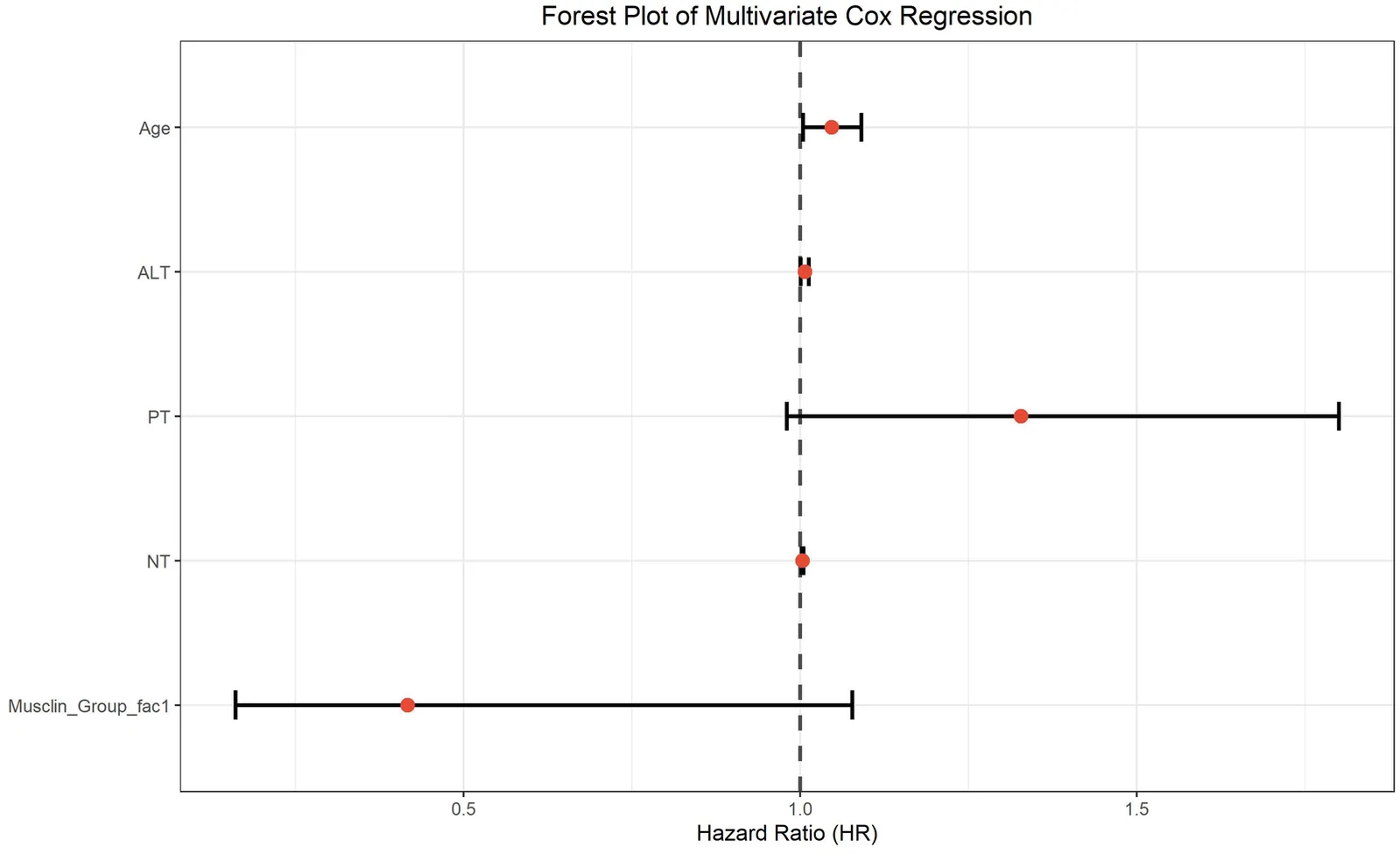
Forest plot of multivariate cox regression for postoperative MACE prediction in AAD patients. Independent predictors included age, ALT, PT, NT-proBNP and dichotomized Musclin (cutoff = 4.815 ng/mL). An HR below 1 indicates an association with a lower event hazard, whereas an HR above 1 indicates an association with a higher event hazard; horizontal lines represent 95% confidence intervals. MACE, major adverse cardiovascular events; AAD, acute aortic dissection; ALT, alanine aminotransferase; NT-proBNP, N-terminal pro-B-type natriuretic peptide.

**Figure 4:**
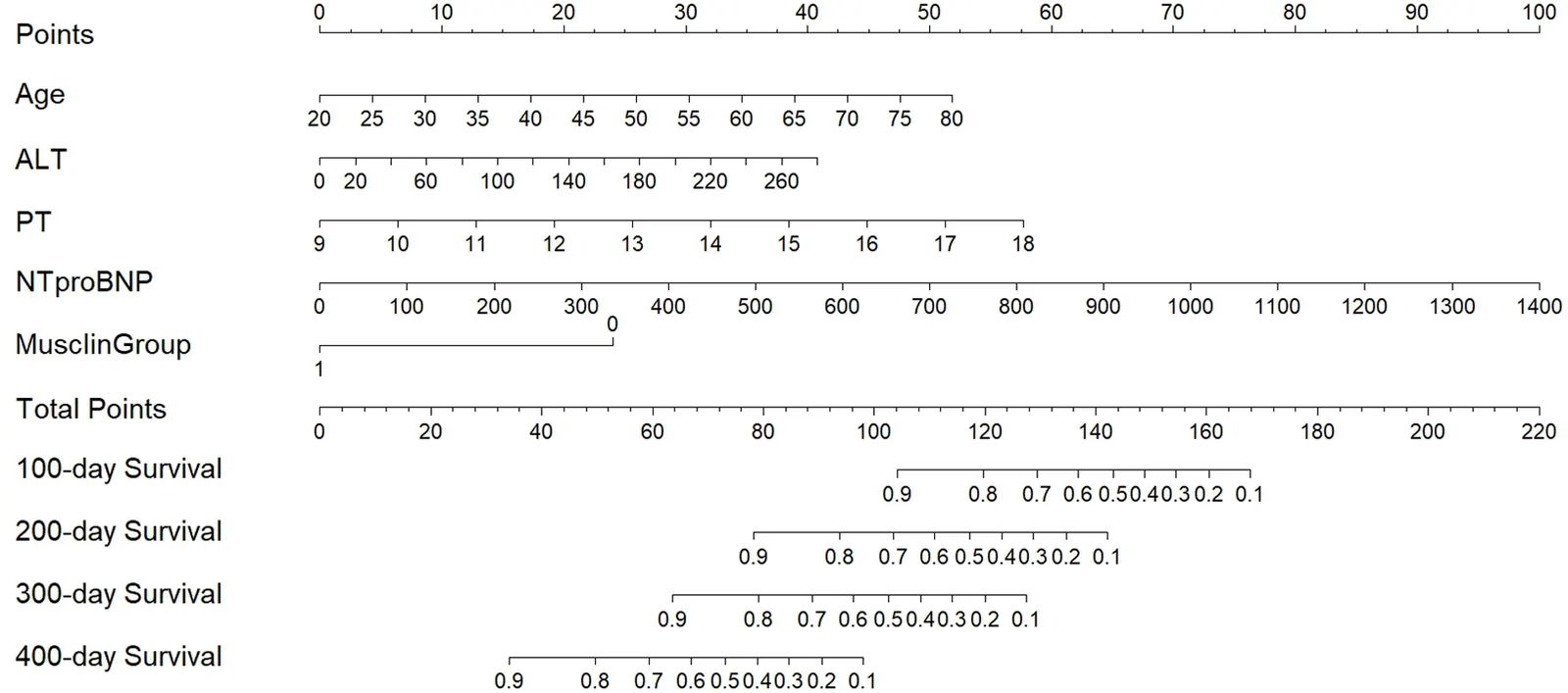
Prognostic nomogram for predicting 100-day, 200-day, 300-day and 400-day MACE-free survival after surgical AAD repair. The nomogram was constructed based on five independent prognostic factors derived from multivariate Cox regression. The total point score can be converted to individual 100-day, 200-day, 300-day, and 400-day MACE-free survival probability.

### Incremental predictive value of circulating musclin beyond conventional clinical model

To determine whether circulating Musclin yields additional prognostic information independent of established conventional risk factors, we comprehensively compared the overall model fit, time-dependent discriminative capacity and risk reclassification performance between Model 1 (clinical model) and Model 2 (clinical model + Musclin). Goodness-of-fit analysis revealed that the −2 log-likelihood (−2LL) value was 253.887 for Model 1 and decreased to 220.586 after the inclusion of Musclin in Model 2, indicating that adding Musclin markedly improved global model fitting.

Time-dependent AUC curves were generated to evaluate dynamic discrimination across the entire follow-up period. For the clinical-only model, the AUC rose gradually from 0.656 at day 50 to a peak of 0.869 at day 350. Meanwhile, the AUC of the Musclin-combined model exhibited a sustained upward trend over time (Figure 5A). The incremental gain in discrimination (*Δ*AUC) conferred by Musclin varied dynamically throughout follow-up: the largest improvements were observed at day 100 (*Δ*AUC = 0.188), whereas the minimal *Δ*AUC of 0.039 was detected at day 250, when the clinical model showed relatively high discrimination. Even at day 250, the combined model still achieved a higher AUC than the clinical model (0.876 vs. 0.837). To evaluate the overall discriminatory ability throughout the entire follow-up period, we plotted the ROC curve at 250 days and compared the differences in AUC using the DeLong test. The DeLong test results showed that at the point where *Δ*AUC was the lowest (250 days), Musclin significantly improved the model performance (*p* = 0.013), indicating that Musclin maintained its ability to distinguish throughout the entire follow-up period (Figure 5B).

**Figure 5:**
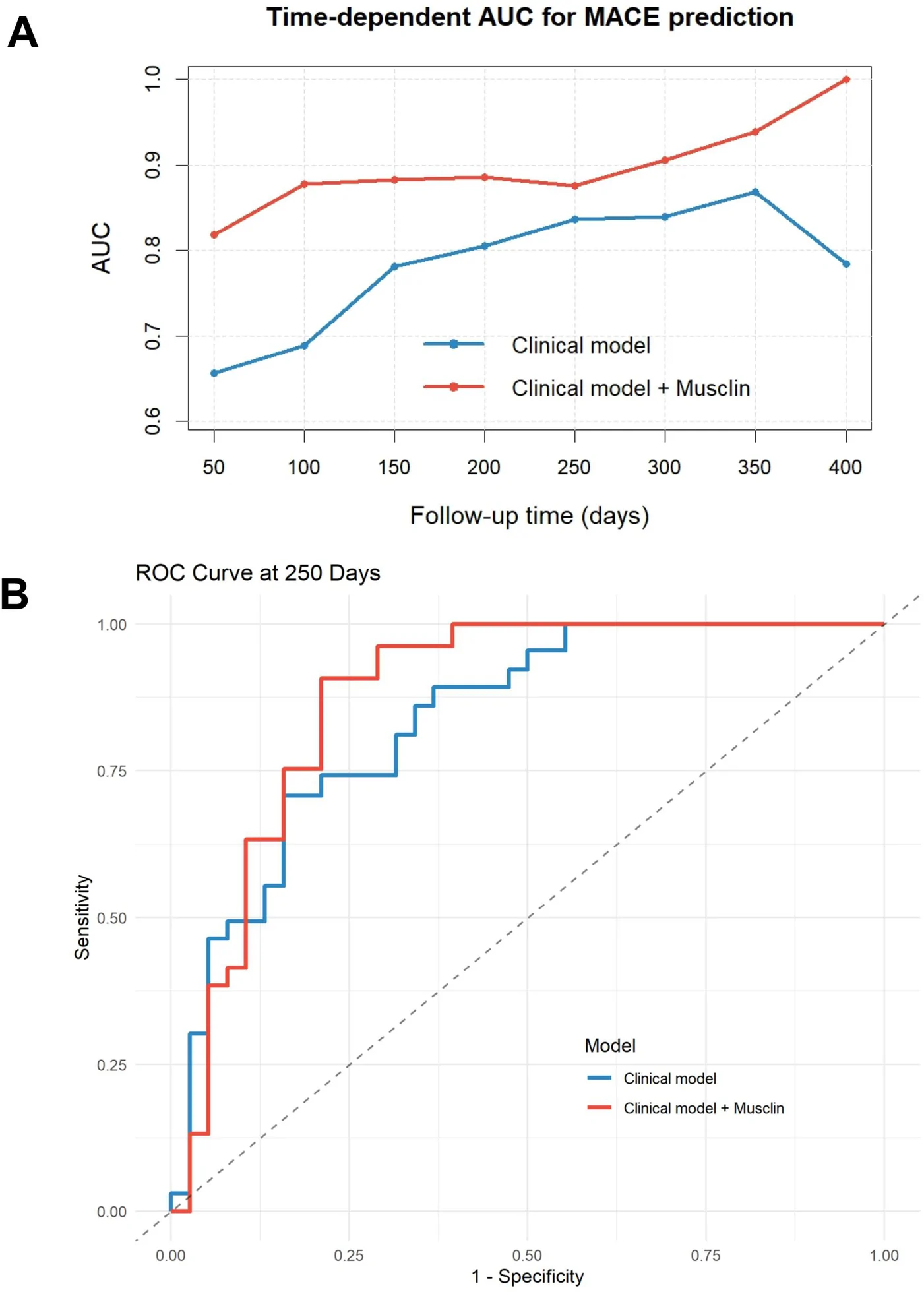
Comparison of discriminative performance between the clinical risk model and musclin-combined model for predicting MACE. **(A)** Time-dependent AUC curves. The blue line represents the conventional clinical model only; the red line denotes the clinical model incorporated with circulating Musclin. The Musclin-augmented model exhibited continuously improved discriminatory ability over time and maintained higher AUC values throughout follow-up. **(B)** Static ROC curves at the 250-day landmark time point. The combined model with Musclin achieved superior predictive accuracy compared with the clinical-only model. DeLong's test confirmed a statistically significant difference in AUC between the two models at 250-day landmark.

Integrated discrimination improvement (IDI) and continuous net reclassification improvement (NRI) were calculated at the 250-day landmark time to quantify gains in risk discrimination and reclassification. After incorporating Musclin into the conventional clinical model, the IDI was 0.153 (95% CI: 0.018–0.286, *P* = 0.012), and the overall NRI was 0.167 (95% CI: 0.003–0.3362, *P* = 0.044). Notably, the event-specific NRI among patients who developed MACE reached 0.361 (*P* = 0.012), suggesting that Musclin substantially enhanced identification of individuals at high risk of MACE and optimized overall risk stratification and individual risk reclassification. In this cohort, adding Musclin improved IDI and NRI estimates at the 250-day landmark; however, these apparent improvements require confirmation with internal resampling and external validation.

### Sensitivity analysis

To verify the robustness of the main analysis results, this study conducted two sensitivity analyses.

First, to verify the robustness of the cutoff value (4.815 ng/mL) determined by the CART decision tree, patients were reclassified into low and high groups using the median Musclin value (4.72 ng/mL) as an alternative threshold, and the Kaplan–Meier survival analysis was repeated. The results showed that the 400-day cumulative incidence of MACE in the low Musclin group remained significantly higher than that in the high Musclin group (log-rank *P* < 0.001), consistent with the main analysis results.

Second, to verify the robustness of Musclin in the multivariate Cox regression model, we retained Musclin as a continuous variable and performed repeated multivariate Cox regression analyses accordingly. The results showed that low Musclin remained independently associated with MACE risk (HR = 0.706, 95% CI 0.553–0.900, *P* < 0.005) (Table 4), further supporting the reliability of the main analysis conclusions.

**Table 4:** Multivariate cox regression analysis incorporating continuous circulating musclin for prediction of MACE.

Variable	β	SE	Wald χ^2^	df	*P* value	HR	95% CI for HR
Age	0.043	0.021	4.156	1	0.041	1.044	1.002–1.088
ALT	0.007	0.003	5.506	1	0.019	1.007	1.001–1.013
PT	0.280	0.155	3.279	1	0.070	1.323	0.977–1.791
NT-proBNP	0.003	0.001	12.854	1	<0.001	1.003	1.001–1.004
Musclin	−0.349	0.124	7.866	1	0.005	0.706	0.553–0.900

ALT, alanine aminotransferase; PT, Prothrombin time; NT-proBNP, N-terminal pro-B-type natriuretic peptide.

## Discussion

In this retrospective cohort of 108 surgically treated AAD patients, lower admission Musclin was associated with subsequent MACE during 400 days of follow-up. Musclin was also inversely correlated with NT-proBNP and positively correlated with ejection fraction, suggesting that it may reflect cardiac functional status and overall illness severity. Although adding Musclin improved apparent model performance in this cohort, these results are hypothesis-generating because the sample was small, the cutoff and model were assessed in the development cohort, and relevant operative covariates were not included.

The biological plausibility of our findings is supported by the emerging role of Musclin as a cardioprotective myokine. Musclin binds to the natriuretic peptide clearance receptor NPR3, thereby reducing the clearance and increasing the bioavailability of natriuretic peptides (10). Experimental studies further suggest that skeletal muscle-derived Musclin protects against pathological cardiac remodeling through natriuretic peptide/cGMP-related signaling, with beneficial effects on systolic function, myocardial fibrosis, and inflammatory responses (5, 11). Our observation that reduced admission Musclin portends worse postoperative cardiovascular outcomes is therefore mechanistically consistent with the loss of these pleiotropic protective pathways in high-risk AAD patients.

Beyond its direct cardiac effects, Musclin appears to exert critical regulatory actions on vascular homeostasis that are particularly relevant to the pathophysiology of aortic dissection. The initiation and propagation of AAD are fundamentally driven by vascular smooth muscle cell (VSMC) apoptosis, phenotypic switching, excessive matrix metalloproteinase (MMP) activation and progressive degradation of aortic extracellular matrix (ECM) (12). Preclinical evidence suggests that Musclin can modulate calcium channel activity, VSMC proliferation and migration, and endothelial–monocyte adhesion (5, 6). Thus, reduced circulating Musclin may reflect impaired endogenous protection against vascular remodeling and inflammation, although this potential mechanism requires direct experimental validation in AAD.

Our findings also support a possible link between skeletal muscle status and cardiovascular outcomes. Sarcopenia and impaired skeletal muscle function have been associated with adverse outcomes after AAD and cardiac surgery (13, 14). Because Musclin is predominantly secreted by skeletal myocytes and its circulating levels are intimately tied to skeletal muscle mass and metabolic health (4), circulating Musclin may provide biochemical information on the skeletal muscle–cardiovascular axis. Low Musclin could therefore represent, at least in part, reduced muscle-related cardiovascular reserve, complementing conventional markers of cardiac stress and systemic injury.

Current biomarker-based risk assessment in AAD mainly relies on indicators of cardiac strain, inflammation, and coagulation, including NT-proBNP, cardiac troponins, C-reactive protein, D-dimer, and fibrinogen (15, 16).While these markers effectively reflect cardiac overload, hemolysis and systemic inflammatory injury, they do not specifically address the skeletal muscle-vascular crosstalk axis. In our cohort, adding Musclin to the clinical model was associated with improved risk discrimination and reclassification at the 250-day landmark, with an IDI of 0.153 and an overall NRI of 0.167; the event-specific NRI was 0.361 (17). These findings suggest that Musclin may provide complementary prognostic information beyond conventional clinical indicators, although its incremental value requires confirmation in larger, independently validated cohorts.

The CART-derived cutoff of 4.815 ng/mL should be interpreted as an exploratory, cohort-specific threshold. Because the cutoff was selected and evaluated in the same small dataset, its apparent separation of risk groups is susceptible to optimism and overfitting. Analyses using the cohort median and continuous Musclin values produced directionally consistent findings, but these sensitivity analyses do not constitute independent validation of the CART threshold. Accordingly, the threshold should not be used for clinical decision-making until it has been evaluated in adequately powered external cohorts.

Several limitations warrant consideration. First, this single-center retrospective study enrolled only 108 patients, which may constrain the generalizability of our findings to centers with differing surgical protocols and patient demographics. Second, despite multivariable adjustment for preoperative clinical and laboratory factors, we did not adjust for critical intraoperative variables, including surgical procedure type, total operation duration, cardiopulmonary bypass duration, aortic cross-clamp time, and intraoperative blood loss. These factors are well-established determinants of postoperative outcomes and may confound the association between Musclin and MACE. Their omission from the current analysis is a notable limitation, and future studies should incorporate these variables to better isolate the independent prognostic value of Musclin. Third, we did not directly quantify skeletal muscle mass or function; therefore, whether the prognostic value of Musclin is mediated through sarcopenia or reflects independent biological information remains to be clarified. Fourth, Musclin was measured retrospectively from archived frozen serum samples; although standardized cryopreservation, blinded testing and duplicate replicates were employed to ensure reliability, subtle pre-analytical variations related to long-term storage cannot be entirely ruled out. Fifth, the CART-derived cutoff was selected and evaluated in the same cohort and may therefore be subject to optimism and overfitting; external validation is required before clinical application. Finally, follow-up was censored at 400 days, and longer-term prognostic utility beyond this horizon requires further investigation.

Notwithstanding these constraints, our findings carry tangible clinical implications. Current preoperative risk stratification in AAD relies on conventional clinical, laboratory and imaging parameters that, while informative, do not specifically gauge skeletal muscle–vascular protective reserve. Circulating Musclin can be readily quantified by a standardized single-point ELISA at admission and integrated into existing perioperative workflows without additional patient burden. If validated in future prospective studies, Musclin measurement could potentially complement established clinical indicators for postoperative risk stratification and surveillance. Future mechanistic studies are warranted to determine whether Musclin is merely a marker of disease severity or plays a causal role in cardiovascular and aortic remodeling. Given the single-center design and modest sample size, our results should be interpreted as hypothesis-generating. The clinical applicability of Musclin measurement for routine risk stratification awaits confirmation in larger, multi-center prospective cohorts with independent validation.

## Conclusion

In this single-center retrospective cohort, lower admission Musclin was associated with a higher risk of postoperative MACE after adjustment for the available clinical variables. Musclin may reflect cardiac dysfunction and overall illness severity, but the study does not establish a causal protective role or definitive independent clinical utility. The CART-derived cutoff and the apparent incremental model performance are exploratory and require prospective validation in independent, adequately powered multicenter cohorts with comprehensive perioperative adjustment.

## Data Availability

The raw data supporting the conclusions of this article will be made available by the authors, without undue reservation.
